# Diagnosis of Sickness in the Street

**Published:** 1890-02-01

**Authors:** 


### DIAGNOSIS OF SICKNESS IN THE STREET.

Some months back, a contributor to The Hospital detailed several cases observed by him of mal-treatment of persons meeting with accidents or taken ill in the streets; the mal-treatment not being intentional, but entirely the result of ignorance of those anxious to assist. There is no doubt that many a person, unconscious from other causes, has been locked up as drunk. Neither is the diagnosis between, for instance, "dead" drunkenness, poisoning by opium, and apoplexy, always clear at a glance, even to a medical man. Dr. A. Rose, of New York, makes a suggestion in a recent number of the New York *Medical Record*. He details the case of an epileptic charged with drunkenness, and imprisoned for ten days. Dr. Rose says: "From what I have seen myself, and what has been seen by others with whom I have spoken on the subject, it appears that some policemen, when they find a person unconscious in the street, never think for a moment that there could possibly be any other cause of unconsciousness but drunkenness." Dr. Rose therefore suggests that medals should be distributed, to be worn by epileptics; "these medals should be familiar to every policeman; there should be a certain mode of attachment, and a certain part of the body where they would have to be looked for (for instance, they might be attached to an indiarubber band round the neck), and by means of these medals policemen would be able to distinguish an unconscious epileptic from a drunkard." Now, the suggestion is good enough so far as it goes. But the unconscious from epileptic fits form but a small portion of the numbers who are by mistake taken away as drunk. Epilepsy is characterised by much struggling, and is least likely to be mistaken for drunkenness than some other maladies. It would be better to forbid confirmed epileptics going about the streets at all without the company of some friend or attendant.

